# Leg Muscle Volume, Intramuscular Fat and Force Generation: Insights From a Computer‐Vision Model and Fat‐Water MRI

**DOI:** 10.1002/jcsm.13735

**Published:** 2025-02-19

**Authors:** Andrew C. Smith, Javier Muñoz Laguna, Eddo O. Wesselink, Zachary E. Scott, Hazel Jenkins, Wesley A. Thornton, Marie Wasielewski, Jordan Connor, Scott Delp, Akshay S. Chaudhari, Todd B. Parrish, Sean Mackey, James M. Elliott, Kenneth A. Weber

**Affiliations:** ^1^ Physical Therapy Program, Department of Physical Medicine and Rehabilitation, School of Medicine University of Colorado Aurora Colorado USA; ^2^ EBPI‐UWZH Musculoskeletal Epidemiology Research Group University of Zurich and Balgrist University Hospital Zurich Switzerland; ^3^ Epidemiology, Biostatistics and Prevention Institute (EBPI) University of Zurich Zurich Switzerland; ^4^ University Spine Centre Zurich (UWZH) Balgrist University Hospital and University of Zurich Zurich Switzerland; ^5^ Faculty of Behavioural and Movement Sciences, Amsterdam Movement Sciences Vrije Universiteit Amsterdam Amsterdam The Netherlands; ^6^ Department of Anesthesiology, Perioperative and Pain Medicine Stanford University School of Medicine Palo Alto California USA; ^7^ Rehabilitation Care Services VA Puget Sound Health Care System Tacoma Washington USA; ^8^ Department of Chiropractic Macquarie University Sydney New South Wales Australia; ^9^ Department of Physical Therapy and Human Movement Sciences, Feinberg School of Medicine Northwestern University Chicago Illinois USA; ^10^ Department of Bioengineering and Mechanical Engineering Stanford University Palo Alto California USA; ^11^ Department of Radiology Stanford University School of Medicine Palo Alto California USA; ^12^ Department of Radiology Northwestern University Chicago Illinois USA; ^13^ Northern Sydney Local Health District The Kolling Institute St. Leonards New South Wales Australia; ^14^ The Faculty of Medicine and Health The University of Sydney Camperdown New South Wales Australia

**Keywords:** computer‐assisted, image processing, leg, magnetic resonance imaging, muscle strength, rehabilitation

## Abstract

**Background:**

Maintaining skeletal muscle health (i.e., muscle size and quality) is crucial for preserving mobility. Decreases in lower limb muscle volume and increased intramuscular fat (IMF) are common findings in people with impaired mobility. We developed an automated method to extract markers of leg muscle health, muscle volume and IMF, from MRI. We then explored their associations with age, body mass index (BMI), sex and voluntary force generation.

**Methods:**

We trained (*n* = 34) and tested (*n* = 16) a convolutional neural network (CNN) to segment five muscle groups in both legs from fat‐water MRI to explore muscle volume and IMF. In 95 participants (70 females, 25 males, mean age [standard deviation] = 34.2 (11.2) years, age range = 18–60 years), we explored associations between the CNN measures and age, BMI and sex, and then in a subset of 75 participants, we explored associations between CNN muscle volume, CNN IMF and maximum plantarflexion force after controlling for age, BMI and sex.

**Results:**

The CNN demonstrated high test accuracy (Sørensen–Dice index ≥ 0.87 for all muscle groups) and reliability (muscle volume ICC_2,1_ ≥ 0.923 and IMF ICC_2_,_1_ ≥ 0.815 for all muscle groups) compared to manual segmentation. CNN muscle volume was positively associated with BMI across all muscle groups (*p* ≤ 0.001) but not with age (*p* ≥ 0.406). CNN IMF was positively associated with age for all muscle groups (*p* ≤ 0.015), and CNN IMF was positively associated with BMI for all muscle groups (*p* ≤ 0.043) except the right deep posterior compartment (*p* = 0.130). Males had greater CNN volume of all muscle groups (*p* < 0.001) except the left and right gastrocnemius (*p* ≥ 0.067). Gastrocnemius CNN IMF was greater in females (*p* ≤ 0.043). Plantarflexion force was positively associated with lateral compartment, soleus and gastrocnemius CNN volume (*p* ≤ 0.025) but not with CNN IMF (*p* ≥ 0.358).

**Conclusions:**

Computer‐vision models combined with fat‐water MRI permits the non‐invasive, automatic assessment of leg muscle volume and IMF. Associations with age, BMI and sex are important when interpreting these measures. Markers of leg muscle health may enhance our understanding of the relationship between muscle health, force generation and mobility.

**Trial Registration:**

ClinicalTrials.gov identifier: NCT02157038

**Abbreviations**
BMI: body mass index
CNN: convolutional neural network
IMF: intramuscular fat
MRI: magnetic resonance imaging

## 1 Introduction

Impairments in mobility are common in many musculoskeletal, neurological and neuromuscular conditions, as well as ageing, negatively impacting one's quality of life [1, 2, 3]. These impairments can range from minor issues to the complete loss of independent ambulation [4]. Normal mobility relies on a complex interaction of multiple systems, including the nervous, musculoskeletal and cardiovascular systems. Dysfunction within or across any of these systems can lead to impaired mobility. Improved markers of mobility may shed light on the pathophysiology of conditions that impair mobility, deliver a deeper understanding of therapeutic mechanisms, improve the tracking of disease progression and recovery and ultimately provide a more comprehensive picture of the interactions between these systems and their specific roles in maintaining mobility across the lifespan.

Maintaining skeletal muscle health (i.e., muscle size and quality) is crucial for preserving mobility. Decreases in muscle size (i.e., muscle atrophy) and changes in lower limb muscle quality are common findings in people with impaired mobility [5]. Muscle size is positively associated with force production [6]. For example, age‐related muscle atrophy in the thigh muscles correlates with reduced knee extension force [7]. Similarly, regarding muscle quality, increases in intramuscular fat (IMF) negatively impact muscle function [8], as seen in ageing individuals where thigh IMF is associated with reduced force production and mobility [9] and in population‐level studies of metabolic disorders [10]. Importantly, declines in skeletal muscle health in conditions affecting mobility can often be reversible with rehabilitation, and improvement in skeletal muscle health often parallels recovery of mobility [11].

Magnetic resonance imaging (MRI) is the gold standard for the volumetric assessment of skeletal muscle health [12]. MRI enables the non‐invasive assessment of the three‐dimensional morphometry of muscle (i.e., muscle size and shape), muscle quality (e.g., IMF) and muscle microstructure (e.g., diffusion tensor imaging) [6, 13]. MRI can provide valuable information on skeletal muscle health, but the extraction of these measures has required manual segmentation of muscle, demanding substantial expertise and time and resulting in subjectivity due to interrater variability. The cost in extracting these measures has been a barrier in their clinical implementation and application in clinical trials [14]. As such, more efficient and objective methods for the quantification of skeletal muscle health are needed to deliver important biomechanical insights into normal human mobility and the muscular mechanisms underlying impairments in mobility.

Recent advances in computer‐vision models, specifically convolutional neural networks (CNNs), allow for automated, rapid and accurate quantification of muscle, removing barriers to the large‐scale assessment of skeletal muscle health. Applications in the cervical spine [15], shoulder [16], lumbar spine [17], hip [18], thigh [19] and leg [19] have been reported. Accordingly, our study aimed to expand on this work and develop an open‐source computer‐vision model using fat‐water MRI to automatically quantify muscle volume and IMF in five muscle groups (anterior compartment, deep posterior compartment, lateral compartment, soleus and gastrocnemius) from the left and right legs (10 muscle groups total). We then explored the relationships between these CNN markers of skeletal muscle health and age, BMI and sex as well as maximum plantarflexion force to develop a foundation for future mechanistic studies and improve our understanding of the relationship between skeletal muscle health, force generation and mobility across the lifespan. We hypothesized that age, BMI and sex would be associated with CNN muscle volume and CNN IMF across all muscle groups and that the lateral compartment, deep posterior compartment, soleus and gastrocnemius CNN muscle volume and CNN IMF would correlate with plantarflexion force.

## 2 Methods

### 2.1 Participants

MRI datasets from 95 participants (70 females, 25 males, mean age [standard deviation] = 34.2 [11.2] years, age range = 18–60 years) were obtained from a prospective observational study exploring longitudinal changes in skeletal muscle health following a whiplash injury due to a motor vehicle collision. Plantarflexion forces were not assessed at the first study time point, so datasets from the second study time point (2 weeks post motor vehicle collision) were used in this study. Inclusion criteria for the original study included 18–65 years of age, Quebec Task Force whiplash grades of II–III, and a primary complaint of neck pain at the first study visit (≤ 1 week post motor vehicle collision), and exclusion criteria included a history of a previous motor vehicle collision, contraindications to MRI, spinal fracture, previous spinal surgery, previous diagnosis of cervical or lumbar radiculopathy and history of neurological or metabolic disorders. The study was approved by Northwestern University's Institutional Review Board, and written informed consent was obtained from every participant. Identifying personal information was removed prior to working with the dataset. See Table 1 for a breakdown of the sample characteristics.

**TABLE 1:** Sample characteristics.

Dataset	*n*	Female (%)	Age (years)	BMI (kg/m^2^)	Left PF (Nm)	Right PF (Nm)
CNN training	34	50.0	31.4 (10.5)	24.8 (4.4)	—	—
CNN testing	16	50.0	33.2 (11.7)	25.6 (3.9)	—	—
Age, BMI and sex	95	73.7	34.2 (11.2)	25.1 (4.5)	—	—
PF	77	72.7	33.6 (10.6)	24.8 (4.5)	121.8 (31.4)	123.2 (27.8)

*Note:* Average (SD).

Abbreviations: BMI = body mass index; CNN = convolutional neural network; PF = plantarflexion force.

### 2.2 Image Acquisition

Imaging was performed supine on a 3.0 T Siemens (Munich, Germany) MAGNETOM Trio scanner using a 6‐channel body array coil placed over the legs. To assess muscle volume and IMF, fat‐water MRI of the bilateral legs was performed using a 3D dual‐echo gradient‐echo FLASH sequence (2‐point Dixon, number of slabs = 1, distance factor = 20%, orientation = transversal, phase encoding direction = anterior–posterior, slice oversampling = 6.7%, slices per slab = 60, field‐of‐view = 320 mm × 190 mm, acquisition matrix = 448 × 266, slice thickness = 5.0 mm, TR = 7.05 ms, TE_1_ = 2.46 ms, TE_2_ = 3.69 ms, averages = 4, flip angle = 12°, in‐plane resolution = 0.71 mm × 0.71 mm, bandwidth = 510 Hz/pixel, GRAPPA acceleration factor = 2, acquisition time = 4 min 24 s). We have previously validated the fat‐water imaging protocol histologically [20].

### 2.3 Manual Muscle Segmentation

In a subset of images from 50 participants balanced by sex, the left and right anterior compartment, deep posterior compartment, lateral compartment, soleus and gastrocnemius (lateral and medial heads) muscle groups were manually segmented in the transverse plane by two independent raters (J. M. L. and H. J.) blinded to demographic and clinical information. We chose a total of 50 participants to be an optimal cut‐off between the time‐consuming burden of manual segmentation while having an equal number of female (*n* = 25) and male participants (*n* = 25) and sufficient variability by age and body mass index (BMI). Tibialis anterior, extensor digitorum longus, extensor hallucis longus and fibularis tertius (if present) were included in the anterior compartment muscle group; tibialis posterior, flexor digitorum longus and flexor hallucis longus were included in the deep posterior compartment muscle group; and fibularis longus and brevis were included in the lateral muscle group. To have a consistent superior border, the raters began the segmentations 4 slices inferior to the superior most aspect of the origin of the tibialis anterior. All raters were doctoral level health professionals with extensive training in lower limb anatomy and musculoskeletal imaging. Segmentation performance between the two raters was assessed using Sørensen‐Dice index, Jaccard index, conformity coefficient, true positive rate, true negative rate, positive predictive value and volume ratio (Table S1). Muscle volume (mL) and IMF were calculated using the segmentation masks from each rater and from the fat‐water imaging. IMF was calculated as the percent of the signal from fat (fat signal divided by the sum of the fat and water signals multiplied by 100).

### 2.4 CNN

We then identified training (*n* = 34) and testing (*n* = 16) datasets matched on age, BMI and sex (Table 1). Using the manual segmentations from both raters as the ground truth, we trained a 2D U‐Net CNN computer‐vision model (spatial window size = 416 × 240; spatial window batch size = 1; channels = 64, 128, 256, 512, and 1026; activation unit = LeakyRelu; strides = 2, 2, 2 and 2; number of residual units = 2; normalization = instance; optimizer = AdamW; loss function = DiceCEloss; weight decay = 1 × 10^−5^; learning rate = 1 × 10^−4^ and batch size = 1) to perform multiclass segmentation of the water images using the open‐source MONAI Python package (Version 0.9.0) based on PyTorch (Version 1.10.2) and a NVIDIA RTX 3090 24 GB graphical processing unit (CUDA Version 12.2, Santa Clara, CA, USA) [21]. We trained the model for 150 000 iterations. The CNN parameters were based on our previous experience [22]. Finally, we applied the trained CNN to the images from all 95 participants.

### 2.5 Plantarflexion Force

Within a subset of the dataset (*n* = 77), we assessed maximum voluntary plantarflexion force as a measure of leg function. Participants were seated in a comfortable position with the hips flexed to 75°, knees flexed to 20° and the ankle positioned in neutral and affixed to an isokinetic dynamometer (Biodex System 3, Shirley, NY, USA). Participants were asked to maximally contract their plantarflexors against the static footplate for 3–4 s while both verbal encouragement and biofeedback via a screen were provided [23]. The maximum plantarflexion force of three trials was used for analysis, and both the left and right leg were assessed.

### 2.6 Statistical Analyses

Using the images from the 50 participants with manual segmentations, interrater accuracy and reliability for muscle volume and IMF between the two manual raters were assessed using correlation and Bland–Altman plots and intraclass correlation coefficients (ICC_2,1_) [24]. In the testing dataset (*n* = 16), we assessed the segmentation performance of the trained CNN to the ground truth from each rater for each muscle, reporting the average segmentation metrics of the two manual raters. Using Bland–Altman plots and intraclass correlation coefficients (ICC_2,1_), we assessed the accuracy and reliability for muscle volume and IMF between the CNN and the ground truth for each muscle using the average muscle volume and IMF of the two manual raters as the ground truth. In a subgroup analysis, we also compared the CNN accuracy between females (*n* = 8) and males (n = 8) in the testing dataset using two‐tailed *t*‐tests. For each muscle, we used multiple linear regression to explore associations between CNN muscle volume and age, BMI and sex. Two‐tailed partial Pearson correlations were used to visualize the independent linear relationship between CNN muscle volume and age for each muscle after controlling for BMI and sex. Likewise, we visualized the relationship between CNN muscle volume and BMI after controlling for age and sex. To explore the relationship between CNN muscle volume and sex for each muscle, we calculated the estimated marginal means of CNN muscle volume for each sex controlling for age and BMI. We then repeated these analyses for CNN IMF. Next, we used hierarchical linear regression to explore the relationship between the corresponding CNN muscle measures (volume and IMF) and plantarflexion force after controlling for age, BMI and sex. Statistical analyses were performed in R (Version 3.5.8) in a Python (Version 3.8.0) environment with the rpy2 (Version 3.5.8) package. Model residuals were tested for normality and homoscedasticity using Shapiro–Wilk tests, P–P plots and scale‐location plots. An α < 0.05 was used as the threshold for statistical significance.

## 3 Results

### 3.1 Interrater Reliability

Interrater reliability for muscle volume was excellent (ICC_2,1_ ≥ 0.774) within the combined training and testing (*n* = 50) datasets for all muscle groups [24]. Interrater reliability for IMF was lower than muscle volume, ranging from fair to excellent (ICC_2,1_ ≥ 0.574). Eroding the segmentations by one iteration (kernel = 3 × 3 × 3), which reduces their volume and increases their specificity, increased the interrater reliability to excellent for all muscle groups (ICC_2,1_ ≥ 0.882). Therefore, the CNN IMF was calculated from the eroded segmentations (see Tables S2–S3, Figures S1–S3).

### 3.2 CNN Accuracy and Reliability

**FIGURE 1:**
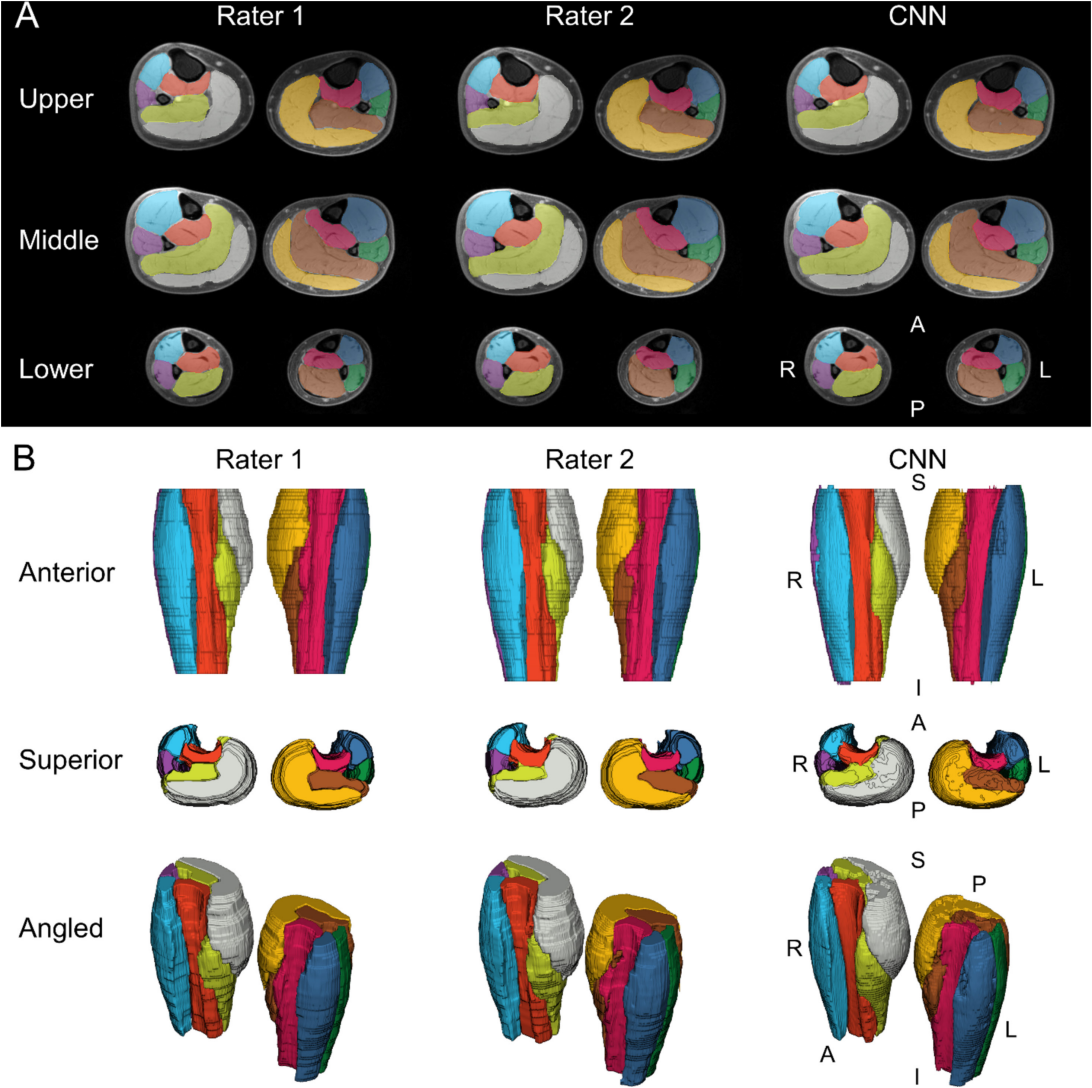
Automatic segmentation of leg muscle groups. Muscle segmentations from Rater 1, Rater 2 and the convolutional neural network (CNN) from a randomly selected testing dataset are shown. (A) Muscle segmentations at the upper, middle and lower leg are overlaid a water image to show changes in muscle morphometry along the superior–inferior axis of the legs. The muscles segmented included the anterior compartment (left = dark blue, right = light blue), deep posterior compartment (left = magenta, right = orange), lateral compartment (left = green, right = purple), soleus (left = brown, right = green‐yellow) and gastrocnemius (left = gold, right = white). L = left, R = right, A = anterior, P = posterior, S = superior, I = inferior.

Within the testing dataset (*n* = 16), the CNN segmentation accuracy was high. The mean Sørensen–Dice index between the CNN and the ground truth was ≥ 0.87 for all muscle groups with a mean volume ratio near 1.00 for all muscle groups (range = 0.98–1.06). See Table S4 for a summary of all segmentation metrics. The CNN accuracy and reliability for muscle volume were high. For all muscle groups, the absolute value of the mean bias in CNN muscle volume was less than 10.0 mL, CNN muscle volume mean absolute error (MAE) was less than 20 mL and CNN muscle volume root mean squared error (RMSE) was less than 25 mL. The CNN reliability for muscle volume was excellent for all muscle groups (ICC_2,1_ ≥ 0.923) (Table S5 and Figure S4). The Bland–Altman plots show some proportional bias with the CNN overestimating small volumes and underestimating large volumes (i.e., regression towards the mean) for the anterior compartment, deep posterior compartment, lateral compartment and gastrocnemius (Figure S4). The CNN accuracy and reliability for IMF was also high. For all muscle groups, the absolute value of the mean bias in CNN IMF was less than 2.0%, CNN IMF MAE was less than 2.0% and CNN IMF RMSE was less than 2.5%. Likewise, the CNN reliability for IMF was good for all muscle groups (ICC_2_,_1_ ≥ 0.815) (Table S5 and Figure S5). Figure 1 compares the ground truth segmentations of the manual raters to the CNN segmentation from a randomly selected testing dataset image. The differences in CNN accuracy between the sexes were small for muscle volume and IMF, only significant for CNN muscle volume in the right anterior compartment (*p* = 0.026) and right lateral compartment (*p* = 0.023), where the CNN overestimated volume in females and underestimated volume in males, and only significant for CNN IMF in the left soleus (*p* = 0.005) and right soleus (*p* = 0.010), where the CNN underestimated IMF in males (Table S6).

### 3.3 Age, Sex and BMI

**FIGURE 2:**
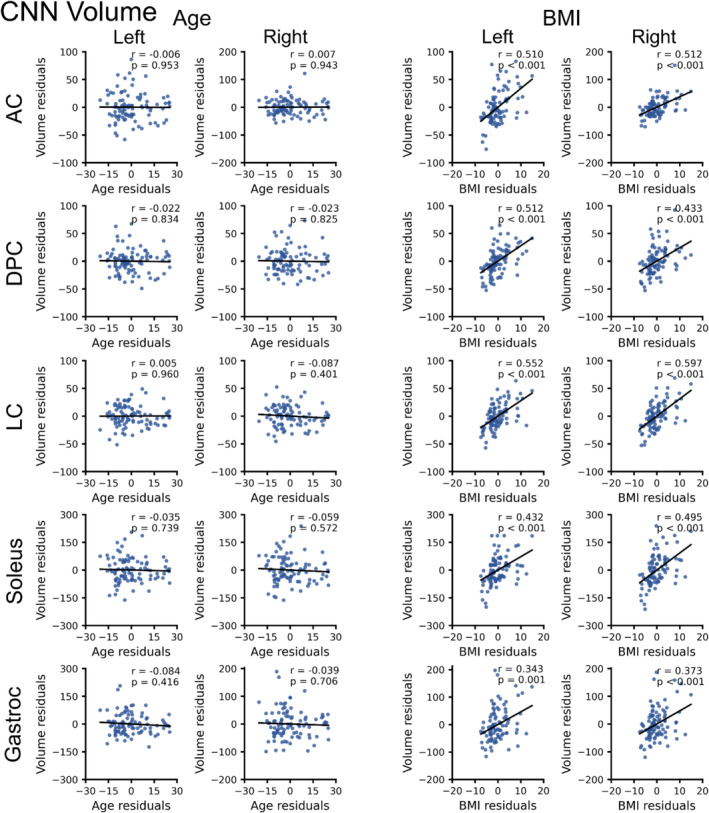
Relationship between convolutional neural network (CNN) muscle volume (mL), age and body mass index (BMI). Partial correlations (Pearson's *r*) were performed to identify linear relationships between CNN volume and age or CNN volume and BMI in 95 participants (70 females, 25 males, age = 34.2 [11.2] years, body mass index [BMI] = 25.1 [4.5] kg/m^2^) after controlling for sex and BMI or sex and age, respectively. For CNN volume and age, the residuals of volume are plotted on the residuals of age after controlling for sex and BMI. For CNN volume and BMI, the residuals of volume are plotted on the residuals of BMI after controlling for sex and age. CNN muscle volume was not associated with age for all muscle groups. CNN muscle volume was positively associated with BMI for all muscle groups. See Table 2 for the results from multiple linear regression analysis with factors of age, BMI and sex. AC = anterior compartment, DPC = deep posterior compartment, LC = lateral compartment, Gastroc = gastrocnemius.

**FIGURE 3:**
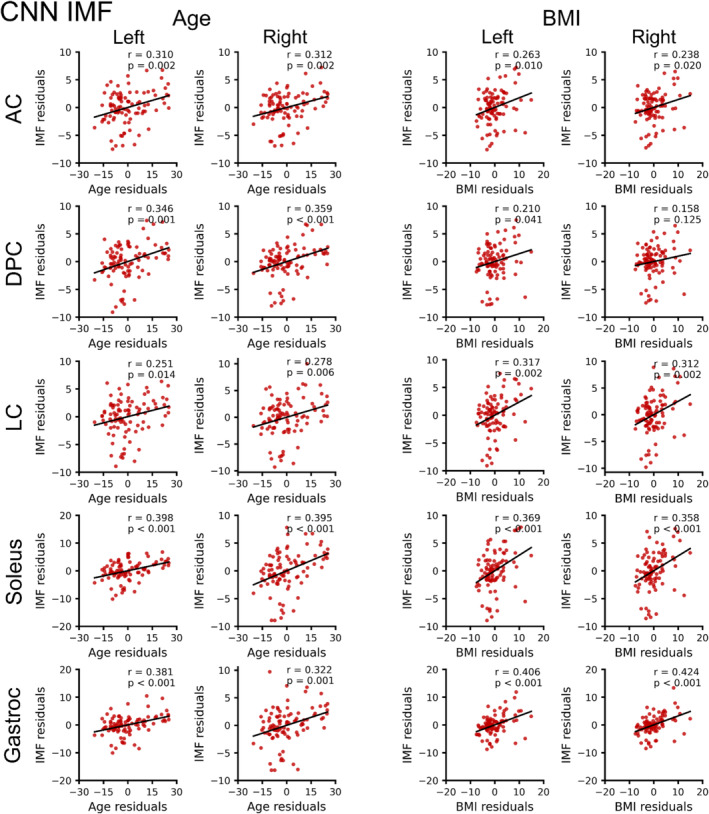
Relationship between convolutional neural network (CNN) intramuscular fat (IMF, %), age and body mass index (BMI). Partial correlations (Pearson's *r*) were performed to identify linear relationships between CNN IMF and age or CNN IMF and BMI in 95 participants (70 females, 25 males, age = 34.2 [11.2] years, BMI = 25.1 [4.5] kg/m^2^). For CNN IMF and age, the residuals of IMF are plotted on the residuals of age after controlling for sex and BMI. For CNN IMF and BMI, the residuals of IMF are plotted on the residuals of BMI after controlling for sex and age. CNN IMF was positively associated with age for all muscle groups. CNN IMF was positively associated with BMI for all muscle groups except the right deep posterior compartment. See Table 2 for the results from multiple linear regression analysis with factors of age, BMI and sex. AC = anterior compartment, DPC = deep posterior compartment, LC = lateral compartment, Gastroc = gastrocnemius.

**FIGURE 4:**
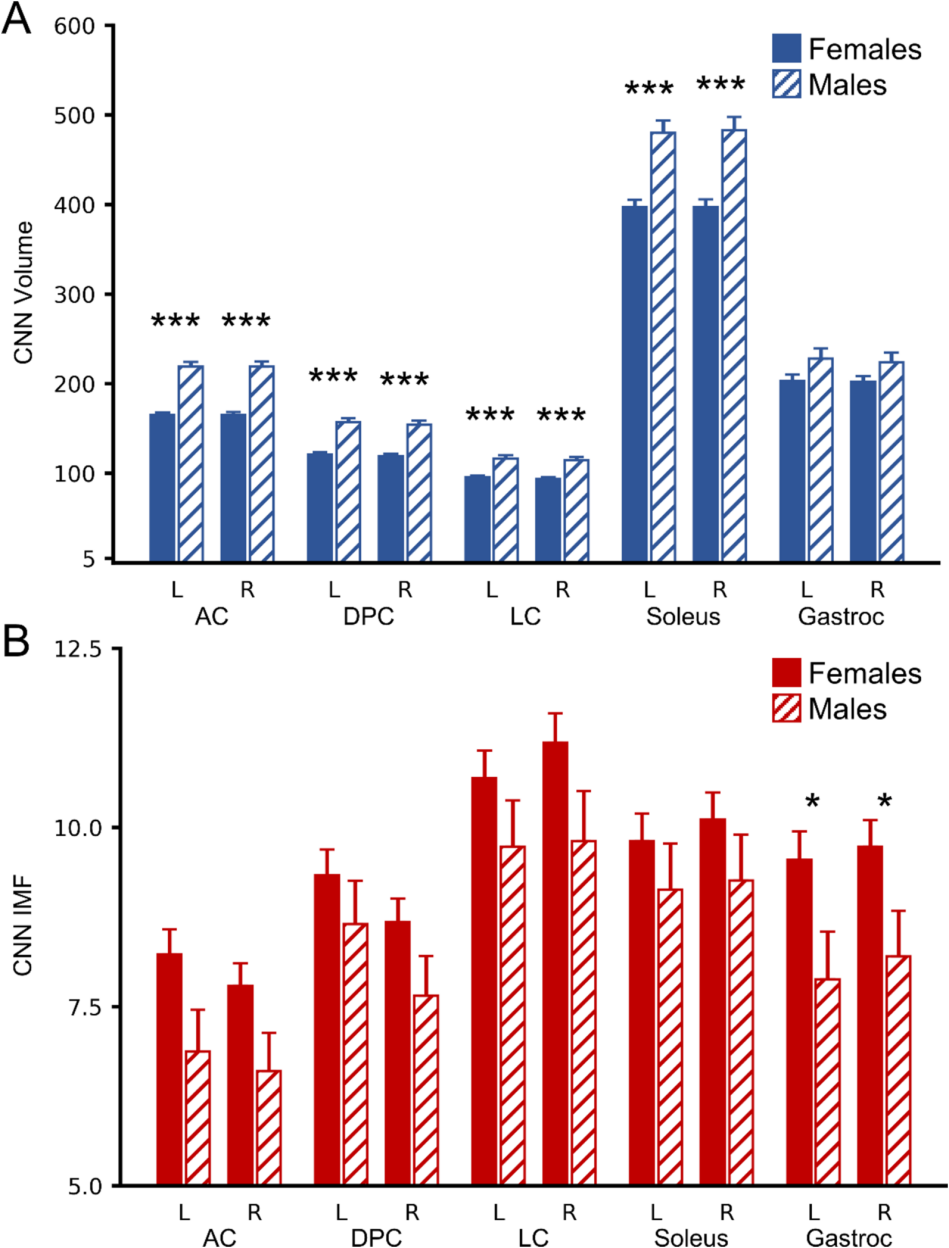
Sex differences in convolutional neural network (CNN) muscle volume (mL) and CNN intramuscular fat (IMF, %) in 95 participants (70 females, 25 males, age = 34.2 (11.2) years, body mass index (BMI) = 25.1 [4.5] kg/m^2^). (A) CNN muscle volume by sex for each muscle. Males had sign larger CNN muscle volume than females for the left and right anterior compartment, deep posterior compartment, lateral compartment, and soleus but not the left and right gastrocnemius (controlling for age and BMI). (B) CNN IMF by sex for each muscle. Females had higher CNN IMF than males for the left and right gastrocnemius (controlling for age and BMI). Estimated marginal means are shown. See Table 2 for results from multiple linear regression with factors of age, BMI and sex. Error bars = 1 standard error. *p < 0.05, ***p < 0.001. AC = anterior compartment, DPC = deep posterior compartment, LC = lateral compartment, Gastroc = gastrocnemius.

Across the entire dataset (*n* = 95), CNN muscle volume was not significantly associated with age for all muscle groups (*p* ≥ 0.406) (Table 2). In contrast, CNN IMF was positively associated with age for all muscle groups (*p* ≤ 0.015). CNN muscle volume was positively associated with BMI for all muscle groups (*p* < 0.001), while CNN IMF was positively associated with BMI for the left and right anterior compartment, left deep posterior compartment, left and right lateral compartment and left and right soleus (*p* ≤ 0.043) but not the right deep posterior compartment (*p* = 0.130). Males had larger CNN muscle volume than females for the left and right anterior compartment, deep posterior compartment, lateral compartment and soleus (*p* < 0.001) but not the left and right gastrocnemius (*p* ≥ 0.067). CNN IMF was higher in females than males for the left and right gastrocnemius (*p* ≤ 0.043) (Figures 2, 3, 4).

**TABLE 2:** Associations with age, BMI and sex (*n* = 95).

CNN volume										
Muscle	Side	Age	*p*	BMI	*p*	Sex	*p*	*F*	*p*	*R* ^2^
AC	Left	−0.004 (0.071)	0.953	0.399 (0.071)	**< 0.001**	0.612 (0.070)	**< 0.001**	38.938	< 0.001	0.562
	Right	0.005 (0.074)	0.944	0.416 (0.073)	**< 0.001**	0.576 (0.073)	**< 0.001**	34.335	< 0.001	0.531
DPC	Left	−0.016 (0.076)	0.836	0.430 (0.075)	**< 0.001**	0.537 (0.075)	**< 0.001**	30.207	< 0.001	0.499
	Right	−0.018 (0.081)	0.827	0.367 (0.080)	**< 0.001**	0.527 (0.080)	**< 0.001**	23.333	< 0.001	0.435
LC	Left	0.004 (0.082)	0.960	0.510 (0.081)	**< 0.001**	0.378 (0.081)	**< 0.001**	22.399	< 0.001	0.425
	Right	−0.066 (0.079)	0.406	0.555 (0.078)	**< 0.001**	0.365 (0.078)	**< 0.001**	26.097	< 0.001	0.462
Soleus	Left	−0.028 (0.086)	0.741	0.389 (0.085)	**< 0.001**	0.437 (0.085)	**< 0.001**	17.218	< 0.001	0.362
	Right	−0.047 (0.084)	0.576	0.451 (0.083)	**< 0.001**	0.413 (0.083)	**< 0.001**	19.847	< 0.001	0.396
Gastroc	Left	−0.080 (0.099)	0.422	0.341 (0.098)	**0.001**	0.181 (0.097)	0.067	5.665	0.001	0.157
	Right	−0.037 (0.098)	0.710	0.373 (0.097)	**< 0.001**	0.164 (0.097)	0.094	6.259	0.001	0.171

CNN IMF										
Muscle	Side	Age	*p*	BMI	*p*	Sex	*p*	*F*	*p*	*R* ^2^
AC	Left	0.296 (0.095)	**0.002**	0.245 (0.094)	**0.011**	−0.187 (0.094)	0.050	8.395	< 0.001	0.217
	Right	0.300 (0.096)	**0.002**	0.222 (0.095)	**0.022**	−0.181 (0.095)	0.059	7.806	< 0.001	0.205
DPC	Left	0.340 (0.097)	**0.001**	0.197 (0.096)	**0.043**	−0.093 (0.095)	0.334	7.175	< 0.001	0.191
	Right	0.354 (0.096)	**< 0.001**	0.146 (0.095)	0.130	−0.151 (0.095)	0.116	7.504	< 0.001	0.198
LC	Left	0.239 (0.097)	**0.015**	0.306 (0.096)	**0.002**	−0.121 (0.095)	0.209	7.253	< 0.001	0.193
	Right	0.263 (0.095)	**0.007**	0.296 (0.095)	**0.002**	−0.158 (0.094)	0.096	8.278	< 0.001	0.214
Soleus	Left	0.371 (0.090)	**< 0.001**	0.337 (0.089)	**< 0.001**	−0.080 (0.089)	0.371	13.245	< 0.001	0.304
	Right	0.369 (0.090)	**< 0.001**	0.326 (0.089)	**< 0.001**	−0.100 (0.089)	0.262	12.932	< 0.001	0.299
Gastroc	Left	0.344 (0.087)	**< 0.001**	0.367 (0.087)	**< 0.001**	−0.184 (0.086)	**0.036**	15.537	< 0.001	0.339
	Right	0.289 (0.089)	**0.002**	0.394 (0.088)	**< 0.001**	−0.181 (0.088)	**0.043**	13.971	< 0.001	0.315

*Note:* Standardized coefficients (standard error). Bold = *p* < 0.050.

Abbreviations: AC = anterior compartment; BMI = body mass index; CNN = convolutional neural network; DPC = deep posterior compartment; Gastroc = gastrocnemius; IMF = intramuscular fat; LC = lateral compartment; *R* ^2^ = variance explained by age, BMI and sex.

### 3.4 Plantarflexion Force

Within a subset of the dataset (*n* = 77) with plantarflexion force measures, the left and right lateral compartment, soleus and gastrocnemius CNN muscle volume was positively associated (after controlling for age, BMI and sex) with left and right plantarflexion force (*p* ≤ 0.025), respectively, with the soleus having the strongest association. Soleus CNN muscle volume along with age, BMI and sex explained about 40%–50% of the variance in plantarflexion force (right *R* ^2^ = 0.399, left *R* ^2^ = 0.483). The additional variance in plantarflexion force explained by CNN muscle volume after controlling for age, BMI, and sex was just greater than 5% for the left (Δ*R* ^2^ = 0.058) and right (Δ*R* ^2^ = 0.063) soleus. CNN IMF was not associated with plantarflexion force for any muscle group (*p* ≥ 0.358) (Table 3).

**TABLE 3:** Associations with plantarflexion force (*n* = 77).

Left plantarflexion force										
Muscle	CNN volume	*F*	*p*	Δ*R* ^2^	*R* ^2^	CNN IMF	*F*	*p*	Δ*R* ^2^	*R* ^2^
Left AC	0.181 (0.133)	1.859	0.177	0.014	0.440	−0.095 (0.102)	0.856	0.358	0.007	0.432
Left DPC	0.032 (0.127)	0.063	0.802	0.001	0.426	−0.035 (0.101)	0.122	0.728	0.001	0.426
Left LC	0.266 (0.113)	5.495	**0.022**	0.041	0.466	−0.053 (0.101)	0.279	0.599	0.002	0.427
Left soleus	0.294 (0.104)	8.051	**0.006**	0.058	0.483	−0.093 (0.106)	0.768	0.384	0.006	0.431
Left gastroc	0.225 (0.093)	5.810	**0.018**	0.043	0.468	−0.100 (0.112)	0.795	0.376	0.006	0.431

Right plantarflexion force										
Muscle	CNN volume	*F*	*p*	Δ*R* ^2^	*R* ^2^	CNN IMF	*F*	*p*	Δ*R* ^2^	*R* ^2^
Right AC	0.208 (0.137)	2.294	0.134	0.020	0.357	0.002 (0.111)	0.000	0.983	< 0.001	0.337
Right DPC	0.037 (0.125)	0.087	0.768	0.001	0.337	−0.024 (0.110)	0.047	0.829	< 0.001	0.337
Right LC	0.300 (0.126)	5.674	**0.020**	0.048	0.385	0.066 (0.107)	0.375	0.542	0.003	0.340
Right Soleus	0.319 (0.116)	7.548	**0.008**	0.063	0.399	0.046 (0.115)	0.164	0.687	0.002	0.338
Right Gastroc	0.230 (0.101)	5.231	**0.025**	0.045	0.381	−0.021 (0.118)	0.032	0.859	< 0.001	0.337

*Note:* Standardized coefficients (standard error). Bold = *p* < 0.050.

Abbreviations: AC = anterior compartment; CNN = convolutional neural network; DPC = deep posterior compartment; Gastroc = gastrocnemius; IMF = intramuscular fat; LC = lateral compartment; ΔR^2^ = additional variance explained by CNN muscle volume or CNN IMF after controlling for age, BMI and sex; *R* ^2^ = variance explained by CNN muscle volume or CNN IMF and age, BMI and sex.

## 4 Discussion

Skeletal muscle health, muscle size and quality, is crucial for maintaining independent mobility. While MRI provides valuable information on skeletal muscle health, the cost in manually extracting MRI‐based measures limits its research and clinical utility. To overcome this, we first trained and tested a CNN computer‐vision model on fat‐water MRI to assess muscle size and IMF in five muscle groups of both legs (10 muscle groups total), demonstrating high test accuracy and reliability. Our analysis showed significant associations between the CNN measures of leg muscle health and age, BMI and sex in 95 participants, and then, in 77 participants, we showed that plantarflexion force was positively associated with lateral compartment, soleus and gastrocnemius CNN muscle volume (controlling for age, BMI and sex) as hypothesized. However, contrary to our hypotheses, plantarflexion force was not associated with deep posterior compartment CNN muscle volume or CNN IMF for any muscle group.

Similar to previous work, we show the feasibility of using computer‐vision models for segmentation of multiple muscle groups allowing for the non‐invasive, automatic assessment of skeletal muscle health with MRI. Our model showed excellent CNN accuracy and reliability for assessing muscle volume and IMF across all leg muscle groups, comparable to models used for the lumbar spine [22], hip [18] and thigh [25] and superior to those for more complex anatomical regions such as the cervical spine [15]. We employed a 2D CNN due to the decreased complexity of the leg muscle anatomy. We have found that 2D models work better in regions where the muscle anatomy is less complex, while 3D models provide improved performance when the spatial relationship of muscles varies significantly across the region (e.g., sternocleidomastoid obliquely traversing the cervical spine) [15, 22]. Our CNN segmentation accuracy (DICE ≥ 0.87 for all muscle groups) was similar to the lower limb segmentation model of Ni et al., which used a 3D CNN [19].

While our reliability between the two human raters was excellent for muscle volume, interrater reliability for IMF was lower initially, ranging from fair to excellent. After eroding the segmentations slightly to reduce their size and increase specificity, the IMF interrater reliability improved to excellent. This was accompanied by a reduction in mean IMF, potentially due to removal of subcutaneous fat near the muscle boundaries, which was erroneously segmented as muscle. This erosion step was included in the CNN IMF calculation pipeline. Alternatively, we could have trained on the eroded masks or used segmentations based on the absolute agreement between the manual raters. Here, however, we chose to train the model using the segmentations of the two raters to improve the generalizability of the model to multiple raters and allow the model to determine the best agreement in a data‐driven manner. Preferably, we would have included segmentations from additional raters, but given the substantial expertise and time associated with manual segmentation, we were limited to two raters. We are now employing model‐assisted manual annotation as used in the *Segment Anything* data engine [26], where we use our pre‐trained models to segment a new image and then have a manual rater correct the segmentation to generate a new annotated ground truth image. Model‐assisted manual annotation is substantially more efficient than full manual segmentation, facilitating the development of large annotated medical imaging datasets.

Information on the impact of age, BMI and sex on leg skeletal muscle health is limited [27, 28]. Therefore, we sought to explore the associations between leg muscle volume and IMF and age, BMI and sex. We found no significant associations between CNN muscle volume and age in any of the leg muscle groups. This is in contrast to other studies, which consistently demonstrate age‐related atrophy across multiple body regions [15, 17]. Findings by Dahlqvist et al. and Fuchs et al. suggest the leg muscles may, considering the daily demands of using the leg muscles, be less susceptible to age‐related muscle loss than other body regions (e.g., thigh muscles), which supports our findings [27, 28]. The lack of significant associations between leg CNN muscle volume and age may also be a feature of the younger age of our sample (mean age = 34.2 years) and the presence of less age‐related atrophy of the leg muscles [29]. We identified weak positive associations between CNN IMF and age in all leg muscle groups, confirming age‐related increases in IMF as previously reported for the leg [27, 28] and other body regions [7, 15, 17, 27]. We found weak positive associations between CNN muscle volume and BMI for all muscle groups in agreement with other reports in the thigh [27] and lumbar spine [17]. We also identified weak to moderate positive associations between CNN IMF and BMI, which reached statistical significance for all muscle groups besides the right deep posterior compartment. This is in contrast to Dahlqvist et al. who found no association between leg (i.e., all muscle groups combined) IMF and BMI but did report significant positive associations between thigh IMF and BMI [27]. Our sample size was almost double (*n* = 95 compared to *n* = 53), providing more power to detect significant associations. Findings have been mixed in other body regions. We previously identified weak positive associations between paraspinal IMF and BMI with the exception of the cervical multifidus, which showed no significant association [15, 17]. Crawford et al. also reported no association between lumbar paraspinal IMF and BMI [27]. The disparate findings may be due to inherent limitations of the broad measure of BMI, which uses mass and height as a crude estimate of healthy weight and does not distinguish between tissue types composing total mass, as well as differences in sample characteristics and the methods used to assess IMF [22]. In agreement with multiple studies, we identified sex differences in muscle size and quality. CNN muscle volume was larger in males than females reaching statistical significance for all muscle groups except the left and right gastrocnemius, and we observed greater CNN IMF in females than males for all muscle groups but only reaching statistical significance for the left and right gastrocnemius. This is consistent with findings showing higher IMF in females than males in the thigh [30], pelvis [31] and other body regions [15, 17, 27]. Sex differences in IMF may be explained by the distribution of muscle fibre types. Females have greater type I/II fibre ratios than males, and type I muscle fibres contain more IMF than type II muscle fibres [32]. Taken together, age, BMI and sex influence muscle size and quality and should be considered when interpreting markers of leg muscle health.

Next, we aimed to link the markers of leg muscle health to leg function by exploring the associations between CNN muscle volume, CNN IMF and plantarflexion force. Because the deep posterior compartment, lateral compartment, soleus and gastrocnemius are plantarflexors, we expected these muscle groups to be associated with plantarflexion force. Consistent with our hypothesis, we found muscle volume of the left and right lateral compartment, soleus and gastrocnemius were positively associated with left and right plantarflexion force, respectively, while, as expected, muscle volume of the anterior compartment was not associated with plantarflexion force. Soleus volume had the strongest association with plantarflexion force. This finding is consistent with the function of the soleus, the largest and primary plantarflexor [33]. As a plantarflexor, we also hypothesized that the deep posterior compartment would be associated with plantarflexion, but no significant association with the deep posterior compartment volume was found, suggesting the deep posterior compartment may be a weaker plantarflexor. The leg muscle volume findings, when taken together, suggest that these may be surrogate markers of voluntary force generation and thus may clinically predict mobility impairments. Contrary to our hypothesis, we found no association between IMF and plantarflexion force. This may also be due to the younger sample age. Perhaps in a sample with greater variance in age and IMF, an association would be present. As age, BMI and sex are associated with strength [7], we used hierarchical linear regression to quantify the variance in plantarflexion force explained by muscle volume and IMF after controlling for age, BMI and sex. Surprisingly, soleus muscle volume only explains an additional 5% of the variance in plantarflexion force after controlling for age, BMI and sex. Soleus volume, age, sex and BMI together explain at most 50% of the variance in plantarflexion force in this sample, leaving a significant amount of the variance in force generation unexplained. Beyond muscle volume and IMF, the distribution of muscle fibre types, type II fibres (i.e., fast twitch) and type I fibres (i.e., slow twitch), also influence the force‐generating capacity of muscle, which we did not measure [34]. That said, the size, quality and composition of muscle only partially explain force generation, as adaptations within the nervous system also have a significant role in force generation (e.g., more complete activation of the motoneuron pool and improved coordination within and between muscles) [35]. Including measures to assess brain, spinal cord and muscle activity related to force generation would likely explain additional variance in the relationship between skeletal muscle health and strength, providing a more comprehensive understanding of the motor control mechanisms related to force generation and mobility across the entire neuromuscular system.

## 5 Limitations

Data were obtained from a prospective observational longitudinal study exploring recovery from a whiplash injury. The images and plantarflexion force measures were from the second study time point, which was 2 weeks following a motor vehicle collision. The muscle volume and IMF measures are not likely to have changed due to disuse or deconditioning during recovery from the injury in this short time frame, and while mobility data were not collected, no differences in plantarflexion force were identified between participants that recovered from the whiplash injury versus having persisting whiplash symptoms at 2 weeks, 3 months or 12 months post motor vehicle collision in the parent study [36]. Therefore, the severity of the whiplash injury was not associated with plantarflexion force. That said, the presence of pain could have reduced the maximum voluntary plantarflexion force [37]. However, neck pain, not leg pain, was the primary complaint, participants had 2 weeks to recover from their injury and the most severe whiplash injuries were excluded (Quebec Task Force whiplash grade IV), mitigating this concern. Next, the associations between muscle volume and plantarflexion force were stronger on the left compared to the right, which may be related to dominant ‘footedness’, as 90% of the sample is likely to be right‐footed [38]. We did not assess footedness, so we were not able to interrogate this further. While the prospective study only collected plantarflexion force, future studies should also examine dorsiflexion as well as knee flexion and extension force to more comprehensively understand how MRI‐based measures of muscle health relate to force generation and mobility. Next, the imaging session also included a comprehensive cervical spine imaging protocol, and due to time constraints, the leg imaging had insufficient resolution and contrast to confidently identify the smaller muscle borders. Therefore, we chose to include and combine the smaller leg muscles into groups with common actions on the ankle joint. Imaging in future studies will be focused on the leg with sufficient image quality to individually segment each muscle of the leg with high confidence. Some participants completed cervical spine fat‐water imaging prior to the leg acquisition, meaning the participants rested supine on the scanner bed between 5 and 30 min prior to starting the fat‐water leg imaging. Moving from standing to supine causes fluid redistribution in the body, which can affect lower limb muscle measures [39]. Having a more consistent supine rest period across the participants could have reduced the variability in the leg measures due to fluid redistribution. Finally, the CNN computer‐vision model showed some proportional bias with muscle volume, overestimating small muscles and underestimating large muscles (i.e., regression towards the mean). This can lead to more stable predictions by dampening the effect of outliers but could also be problematic when needing to accurately assess muscle volume in people on the extremes of body size. It is important to include greater heterogeneity in the sample by age, body size, BMI, ethnicity and race to capture more diversity and improve the accuracy and generalizability of computer‐vision models and their findings to the global population.

Applying computer‐vision models to fat‐water MRI allows for the non‐invasive, automatic assessment of leg muscle health, aiding the integration of these measures in mobility research. When interpreting measures of leg muscle health, the associations with age, BMI and sex should be considered. Including these markers in mechanistic studies and clinical trials may better link skeletal muscle health to mobility, enhancing our ability to recover impaired mobility and maintain it across the lifespan.

## Ethics Statement

We confirm that we have read the journal's position on issues involved in ethical publication and affirm that this report is consistent with those guidelines.

## Conflicts of Interest

James M. Elliott has a 3% interest in Orofacial Therapeutics, LLC and receives royalties (< USD$10 000 annually) for online educational courses regarding trauma informed care and whiplash associated disorders. Akshay S. Chaudhari has provided consulting services to Patient Square Capital and Elucid Bioimaging; has ownership interests in Subtle Medical, Brain Key and LVIS Corp; and receives research support from GE Healthcare, Philips, Amazon, Microsoft and Stability.ai. The information provided in this study are unrelated to those perceived conflicts of interest. All remaining authors declare no conflicts of interest.

## Supporting information


**Table S1** Segmentation performance metrics.
**Table S2.** Interrater segmentation accuracy (*n* = 50).
**Table S3.** Interrater muscle volume and IMF accuracy and reliability (*n* = 50).
**Figure S1.** Interrater accuracy and reliability of muscle volume (mL) between the two manual raters on the training and testing datasets (*n* = 50). Correlation and Bland–Altman plots are shown for each muscle. In the correlation plot, the solid black line represents the best fit line, and the dashed grey line represents perfect agreement (y = x). In the Bland–Altman plots, the dashed black and grey lines indicate the mean difference (i.e., bias) ± 1.96 × standard deviation (i.e., 95% limits of agreement). The solid black line together with the linear regression equation summarize the direction and magnitude of proportional bias. ICC = intraclass correlation coefficient. AC = anterior compartment, DPC = deep posterior compartment, LC = lateral compartment, Gastroc = gastrocnemius.
**Figure S2.** Interrater accuracy and reliability of intramuscular fat (IMF, %) between the two manual raters on the training and testing datasets (*n* = 50) without eroding the segmentations. Correlation and Bland–Altman plots are shown for each muscle. In the correlation plot, the solid black line represents the best fit line, and the dashed grey line represents perfect agreement (y = x). In the Bland–Altman plots, the dashed black and grey lines indicate the mean difference (i.e., bias) ± 1.96 × standard deviation (i.e., 95% limits of agreement). The solid black line together with the linear regression equation summarize the direction and magnitude of proportional bias. ICC = intraclass correlation coefficient. AC = anterior compartment, DPC = deep posterior compartment, LC = lateral compartment, Gastroc = gastrocnemius.
**Figure S3.** Interrater accuracy and reliability of intramuscular fat (IMF, %) between the two manual raters on the training and testing datasets (*n* = 50) after eroding the segmentations. Correlation and Bland–Altman plots are shown for each muscle. In the correlation plot, the solid black line represents the best fit line, and the dashed grey line represents perfect agreement (y = x). In the Bland–Altman plots, the dashed black and grey lines indicate the mean difference (i.e., bias) ± 1.96 × standard deviation (i.e., 95% limits of agreement). The solid black line together with the linear regression equation summarize the direction and magnitude of proportional bias. ICC = intraclass correlation coefficient. AC = anterior compartment, DPC = deep posterior compartment, LC = lateral compartment, Gastroc = gastrocnemius.
**Table S4.** Testing segmentation accuracy (*n* = 16).
**Table S5.** Testing muscle volume and IMF accuracy and reliability (*n* = 16).
**Table S6.** Testing muscle volume and IMF accuracy by sex.
**Figure S4.** Accuracy and reliability of the automated assessment of muscle volume (mL) with respect to manual segmentation on the testing dataset (*n* = 16). Correlation and Bland–Altman plots are shown for each muscle. In the correlation plot, the solid black line represents the best fit line, and the dashed grey line represents perfect agreement (y = x). In the Bland–Altman plots, the dashed black and grey lines indicate the mean difference (i.e., bias) ± 1.96 × standard deviation (i.e., 95% limits of agreement). The solid black line together with the linear regression equation summarize the direction and magnitude of proportional bias. ICC = intraclass correlation coefficient, CNN = convolutional neural network, GT = ground truth. AC = anterior compartment, DPC = deep posterior compartment, LC = lateral compartment, Gastroc = gastrocnemius.
**Figure S5.** Accuracy and reliability of the automated assessment of intramuscular fat (IMF, %) with respect to manual segmentation on the testing dataset (*n* = 16). Correlation and Bland–Altman plots are shown for each muscle. In the correlation plot, the solid black line represents the best fit line, and the dashed grey line represents perfect agreement (y = x). In the Bland–Altman plots, the dashed black and grey lines indicate the mean difference (i.e., bias) ± 1.96 × standard deviation (i.e., 95% limits of agreement). The solid black line together with the linear regression equation summarize the direction and magnitude of proportional bias. ICC = intraclass correlation coefficient, CNN = convolutional neural network, GT = ground truth. AC = anterior compartment, DPC = deep posterior compartment, LC = lateral compartment, Gastroc = gastrocnemius.

## Data Availability

The de‐identified datasets used in this study are available from the corresponding author upon reasonable request. The computer‐vision segmentation model was developed using open‐source Python packages (Pytorch and MONAI). The computer‐vision model and associated scripts are openly available on GitHub for transparency, replication, reproduction and further research in more diverse samples (https://github.com/MuscleMap/MuscleMap/).
