# Supplementary material for: Leg Muscle Volume, Intramuscular Fat and Force Generation: Insights From a Computer‐Vision Model and Fat‐Water MRI
Source: J Cachexia Sarcopenia Muscle. 2025 Feb 19;16(1):e13735. doi: 10.1002/jcsm.13735 (PMC11839747; doi:10.1002/jcsm.13735)
Supplement: Supplementary file 1 — Table S1 Segmentation performance metrics. Table S2. Interrater segmentation accuracy (n = 50). Table S3. Interrater muscle volume and IMF accuracy and reliability (n = 50). Figure S1. Interrater accuracy and reliability of muscle volume (mL) between the two manual raters on the training and testing datasets (n = 50). Correlation and Bland–Altman plots are shown for each muscle. In the correlation plot, the solid black line represents the best fit line, and the dashed grey line represents perfect agreement (y = x). In the Bland–Altman plots, the dashed black and grey lines indicate the mean difference (i.e., bias) ± 1.96 × standard deviation (i.e., 95% limits of agreement). The solid black line together with the linear regression equation summarize the direction and magnitude of proportional bias. ICC = intraclass correlation coefficient. AC = anterior compartment, DPC = deep posterior compartment, LC = lateral compartment, Gastroc = gastrocnemius. Figure S2. Interrater accuracy and reliability of intramuscular fat (IMF, %) between the two manual raters on the training and testing datasets (n = 50) without eroding the segmentations. Correlation and Bland–Altman plots are shown for each muscle. In the correlation plot, the solid black line represents the best fit line, and the dashed grey line represents perfect agreement (y = x). In the Bland–Altman plots, the dashed black and grey lines indicate the mean difference (i.e., bias) ± 1.96 × standard deviation (i.e., 95% limits of agreement). The solid black line together with the linear regression equation summarize the direction and magnitude of proportional bias. ICC = intraclass correlation coefficient. AC = anterior compartment, DPC = deep posterior compartment, LC = lateral compartment, Gastroc = gastrocnemius. Figure S3. Interrater accuracy and reliability of intramuscular fat (IMF, %) between the two manual raters on the training and testing datasets (n = 50) after eroding the segmentations. Correlation and Blan [file JCSM-16-e13735-s001.docx]

**Leg Muscle Volume, Intramuscular Fat, and Force Generation: Insights from a Computer Vision Model and Fat-Water MRI**

**Supplementary Material**

Supplementary Table 1. Segmentation Performance Metrics

| **Metric** | **Equation** | **Range** | **Meaning** |
| --- | --- | --- | --- |
| Sørensen-Dice Index (DICE) | $\frac{2\times\left\vert SM\cap GT \right\vert}{\left\vert SM \right\vert+\left\vert GT \right\vert}$ | 0 – 1 | Spatial overlap  between masks |
| Jaccard Index | $\frac{\left\vert SM\cap GT \right\vert}{\left\vert SM \right\vert+\left\vert GT \right\vert-\left\vert SM\cap GT \right\vert}$ | 0 – 1 | Spatial overlap  between masks |
| Conformity Coefficient | $1-\frac{FP+FN}{TP}$ | ≤ 1 | Ratio of incorrectly and correctly segmented voxels |
| True Positive Rate (TPR) | $\frac{TP}{TP+FN}$ | 0 – 1 | Sensitivity |
| True Negative Rate (TNR) | $\frac{TN}{TN+FP}$ | 0 – 1 | Specificity |
| Positive Predictive Value (PPV) | $\frac{TP}{TP+FP}$ | 0 – 1 | Precision |
| Volume Ratio | $\frac{\left\vert SM \right\vert}{\left\vert GT \right\vert}$ | ≥ 0 | Ratio of mask volumes |

SM = segmentation mask; GT = ground truth mask; TP = true positive, voxels correctly segmented as deep cervical extensor muscle; TN = true negative, voxels correctly segmented as background; FP = false positive, voxels incorrectly segmented as deep cervical extensor muscle, FN = false negative, voxels incorrectly segmented as background. The masks from each of the three raters were used as the GT for the performance metrics.

**Supplementary Table 2. Interrater Segmentation Accuracy (n = 50)**

| **Muscle** | **Side** | **Dice** | **JI** | **CC** | **TPR** | **TNR** | **PPV** | **VR** |
| --- | --- | --- | --- | --- | --- | --- | --- | --- |
| AC | Left | 0.93 (< 0.01) | 0.87 (0.01) | 0.85 (0.01) | 0.91 (0.01) | 1.00 (< 0.01) | 0.96 (< 0.01) | 0.95 (0.01) |
|  | Right | 0.93 (< 0.01) | 0.87 (0.01) | 0.85 (0.01) | 0.91 (0.01) | 1.00 (< 0.01) | 0.95 (0.01) | 0.96 (0.01) |
| DPC | Left | 0.88 (0.01) | 0.78 (0.01) | 0.72 (0.01) | 0.83 (0.01) | 1.00 (< 0.01) | 0.93 (0.01) | 0.90 (0.02) |
|  | Right | 0.88 (0.01) | 0.78 (0.02) | 0.73 (0.02) | 0.85 (0.01) | 1.00 (< 0.01) | 0.92 (0.01) | 0.93 (0.02) |
| LC | Left | 0.90 (0.01) | 0.78 (0.03) | 0.79 (0.02) | 0.88 (0.01) | 1.00 (< 0.01) | 0.93 (0.01) | 0.95 (0.03) |
|  | Right | 0.90 (0.01) | 0.78 (0.04) | 0.78 (0.01) | 0.90 (0.01) | 1.00 (< 0.01) | 0.91 (0.01) | 0.99 (0.02) |
| Soleus | Left | 0.93 (< 0.01) | 0.78 (0.05) | 0.85 (0.01) | 0.92 (0.01) | 1.00 (< 0.01) | 0.95 (0.01) | 0.97 (0.01) |
|  | Right | 0.93 (< 0.01) | 0.78 (0.06) | 0.85 (0.01) | 0.91 (0.01) | 1.00 (< 0.01) | 0.95 (< 0.01) | 0.97 (0.01) |
| Gastroc | Left | 0.89 (0.01) | 0.78 (0.07) | 0.74 (0.02) | 0.86 (0.01) | 1.00 (< 0.01) | 0.92 (0.01) | 0.94 (0.02) |
|  | Right | 0.88 (0.01) | 0.78 (0.08) | 0.73 (0.02) | 0.84 (0.01) | 1.00 (< 0.01) | 0.93 (0.01) | 0.91 (0.02) |

Interrater accuracy of the segmentations between the two manual raters were assessed on the training and testing datasets (n = 50) using the Sørensen-Dice index (Dice), Jaccard index (JI), conformity coefficient (CC), true positive rate (TPR), true negative rate (TNR), positive predictive value (PPV), and volume ratio (VR). Metrics shown = average (standard error). AC = anterior compartment, DPC = deep posterior compartment, LC = lateral compartment, Gastroc = gastrocnemius.

**Supplementary Table 3. Interrater Muscle Volume and IMF Accuracy and Reliability (n = 50)**

| **Volume (ml)** | | | | | | | | | | |
| --- | --- | --- | --- | --- | --- | --- | --- | --- | --- | --- |
| **Muscle** | **Side** | **Mean** | **Bias** | **95% LA** | **MAE** | **RMSE** | **R^2^** | **ICC** | **95% CI** | **p** |
| AC | Left | 191.9 (6.7) | 10.1 | -10.2 – 30.3 | 11.9 | 14.4 | 0.905 | 0.955 | 0.71 – 0.98 | < 0.001 |
|  | Right | 193.0 (6.6) | 10.2 | -12.6 – 33.1 | 13.1 | 15.5 | 0.891 | 0.947 | 0.74 – 0.98 | < 0.001 |
| DPC | Left | 138.8 (6.0) | 17.2 | -25.2 – 59.7 | 18.2 | 27.7 | 0.395 | 0.774 | 0.38 – 0.90 | < 0.001 |
|  | Right | 136.9 (5.8) | 13.7 | -22.7 – 50.2 | 16.4 | 23.1 | 0.627 | 0.842 | 0.55 – 0.93 | < 0.001 |
| LC | Left | 107.4 (3.6) | 8.0 | -10.1 – 26.0 | 9.6 | 12.2 | 0.761 | 0.892 | 0.57 – 0.96 | < 0.001 |
|  | Right | 106.4 (3.6) | 4.6 | -14.6 – 23.9 | 8.6 | 10.9 | 0.817 | 0.911 | 0.82 – 0.95 | < 0.001 |
| Soleus | Left | 432.5 (13.2) | 3.7 | -46.8 – 54.2 | 20.4 | 26.0 | 0.924 | 0.962 | 0.93 – 0.98 | < 0.001 |
|  | Right | 433.9 (14.1) | 1.8 | -51.8 – 55.5 | 20.8 | 27.4 | 0.925 | 0.963 | 0.94 – 0.98 | < 0.001 |
| Gastroc | Left | 226.3 (10.9) | 21.4 | -25.7 – 68.5 | 27.8 | 32.2 | 0.782 | 0.909 | 0.60 – 0.97 | < 0.001 |
|  | Right | 217.7 (9.9) | 18.3 | -22.8 – 59.4 | 21.5 | 27.8 | 0.808 | 0.918 | 0.64 – 0.97 | < 0.001 |
|  |  |  |  |  |  |  |  |  |  |  |
| **IMF (%)** | | | | | | | | | | |
| **Muscle** | **Side** | **Mean** | **Bias** | **95% LA** | **MAE** | **RMSE** | **R^2^** | **ICC** | **95% CI** | **p** |
| AC | Left | 10.5 (0.4) | 1.8 | -0.7 – 4.2 | 1.9 | 2.2 | 0.400 | 0.774 | -0.01 – 0.93 | < 0.001 |
|  | Right | 10.5 (0.4) | 1.9 | -1.0 – 4.9 | 2.0 | 2.5 | 0.045 | 0.657 | -0.03 – 0.87 | < 0.001 |
| DPC | Left | 11.5 (0.5) | 1.2 | -0.8 – 3.3 | 1.3 | 1.6 | 0.699 | 0.876 | 0.26 – 0.96 | < 0.001 |
|  | Right | 10.5 (0.4) | 1.0 | -0.4 – 2.4 | 1.0 | 1.2 | 0.760 | 0.895 | 0.18 – 0.97 | < 0.001 |
| LC | Left | 13.8 (0.5) | 1.8 | -1.9 – 5.4 | 2.0 | 2.5 | 0.519 | 0.797 | 0.28 – 0.92 | < 0.001 |
|  | Right | 14.3 (0.5) | 1.1 | -2.9 – 5.2 | 1.8 | 2.4 | 0.590 | 0.793 | 0.58 – 0.89 | < 0.001 |
| Soleus | Left | 10.8 (0.4) | 0.6 | -0.3 – 1.6 | 0.7 | 0.8 | 0.936 | 0.969 | 0.57 – 0.99 | < 0.001 |
|  | Right | 10.8 (0.4) | 0.5 | -0.6 – 1.6 | 0.6 | 0.8 | 0.930 | 0.969 | 0.80 – 0.99 | < 0.001 |
| Gastroc | Left | 13.7 (0.5) | 3.0 | -2.2 – 8.3 | 3.5 | 4.0 | 0.003 | 0.574 | -0.02 – 0.82 | < 0.001 |
|  | Right | 13.0 (0.4) | 2.3 | -0.7 – 5.2 | 2.3 | 2.7 | 0.249 | 0.684 | -0.06 – 0.89 | < 0.001 |
|  |  |  |  |  |  |  |  |  |  |  |
| **IMF Eroded (%)** | | | | | | | | | | |
| **Muscle** | **Side** | **Mean** | **Bias** | **95% LA** | **MAE** | **RMSE** | **R^2^** | **ICC** | **95% CI** | **p** |
| AC | Left | 7.5 (0.4) | 0.1 | -0.7 – 1.0 | 0.3 | 0.4 | 0.968 | 0.984 | 0.97 – 0.99 | < 0.001 |
|  | Right | 7.3 (0.3) | 0.1 | -0.8 – 1.0 | 0.3 | 0.5 | 0.955 | 0.978 | 0.96 – 0.99 | < 0.001 |
| DPC | Left | 9.1 (0.4) | 0.7 | -1.0 – 2.4 | 0.8 | 1.1 | 0.817 | 0.921 | 0.71 – 0.97 | < 0.001 |
|  | Right | 8.3 (0.3) | 0.4 | -0.8 – 1.6 | 0.6 | 0.7 | 0.872 | 0.941 | 0.81 – 0.97 | < 0.001 |
| LC | Left | 10.5 (0.5) | 0.4 | -1.9 – 2.7 | 0.8 | 1.2 | 0.845 | 0.925 | 0.86 – 0.96 | < 0.001 |
|  | Right | 11.0 (0.4) | 0.2 | -1.7 – 2.2 | 0.6 | 1.0 | 0.898 | 0.947 | 0.91 – 0.97 | < 0.001 |
| Soleus | Left | 9.3 (0.4) | -0.1 | -0.6 – 0.5 | 0.2 | 0.3 | 0.991 | 0.995 | 0.99 – 1.00 | < 0.001 |
|  | Right | 9.4 (0.4) | -0.1 | -0.6 – 0.4 | 0.2 | 0.3 | 0.989 | 0.995 | 0.99 – 1.00 | < 0.001 |
| Gastroc | Left | 9.2 (0.4) | 0.5 | -2.4 – 3.3 | 0.9 | 1.5 | 0.774 | 0.882 | 0.80 – 0.93 | < 0.001 |
|  | Right | 9.1 (0.4) | 0.2 | -0.6 – 1.0 | 0.4 | 0.5 | 0.971 | 0.985 | 0.97 – 0.99 | < 0.001 |

Interrater accuracy of the segmentations between the two manual raters were assessed on the combined training and testing datasets (n = 50) using Bland-Altman analyses and intraclass correlation coefficients (ICC_2,1_). IMF = intramuscular fat, LA = limits of agreement, MAE = mean absolute error, RMSE = root mean squared error, ICC = intraclass correlation coefficient, CI = Confidence Interval, AC = anterior compartment, DPC = deep posterior compartment, LC = lateral compartment, Gastroc = gastrocnemius.


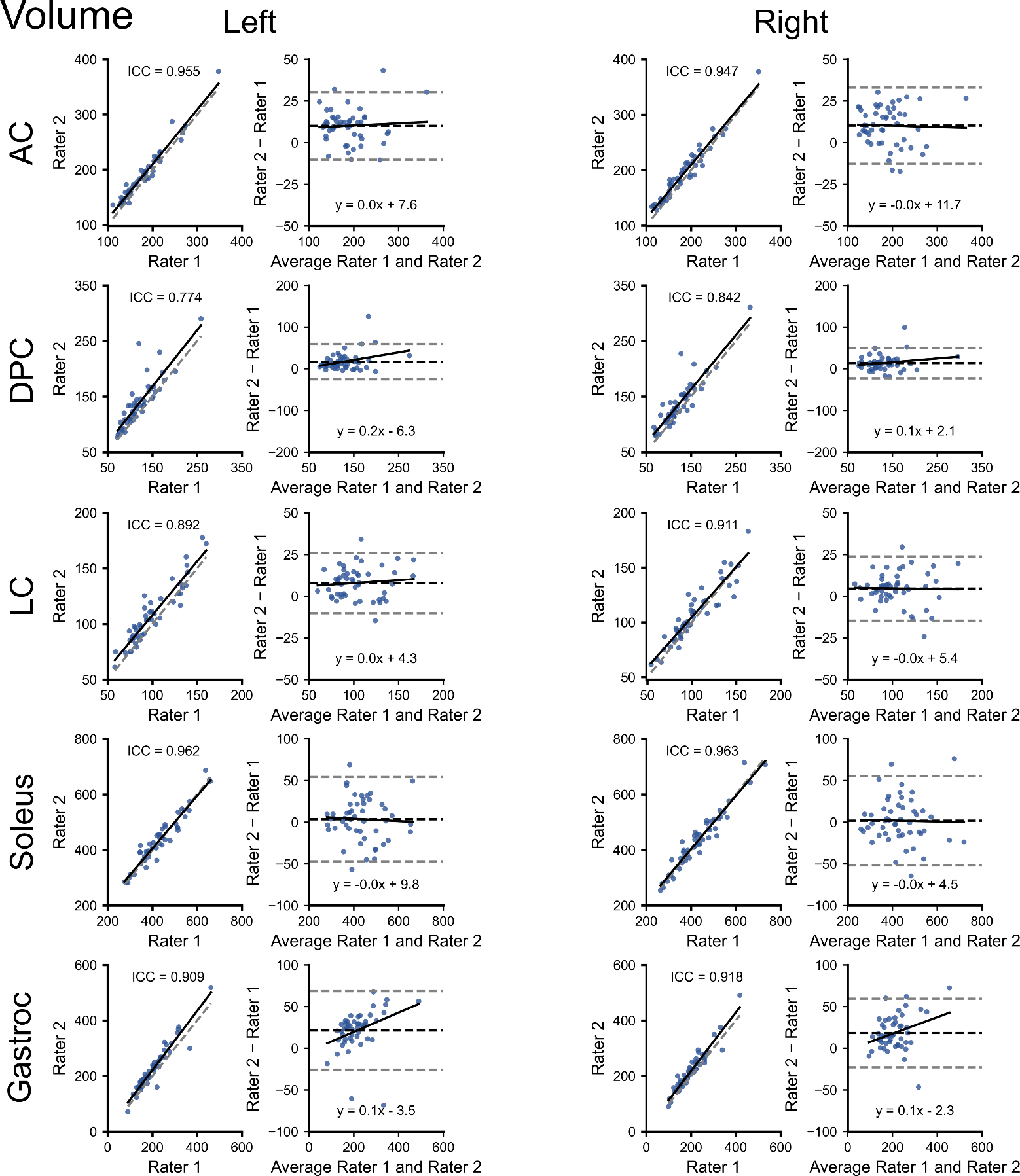


**Supplementary Figure 1.** Interrater accuracy and reliability of muscle volume (ml) between the two manual raters on the training and testing datasets (n = 50). Correlation and Bland-Altman plots are shown for each muscle. In the correlation plot, the solid black line represents the best fit line, and the dashed gray line represents perfect agreement (y = x). In the Bland-Altman plots, the dashed black and gray lines indicate the mean difference (i.e., bias) ± 1.96 × standard deviation (i.e., 95% limits of agreement). The solid black line together with the linear regression equation summarize the direction and magnitude of proportional bias. ICC = intraclass correlation coefficient. AC = anterior compartment, DPC = deep posterior compartment, LC = lateral compartment, Gastroc = gastrocnemius.


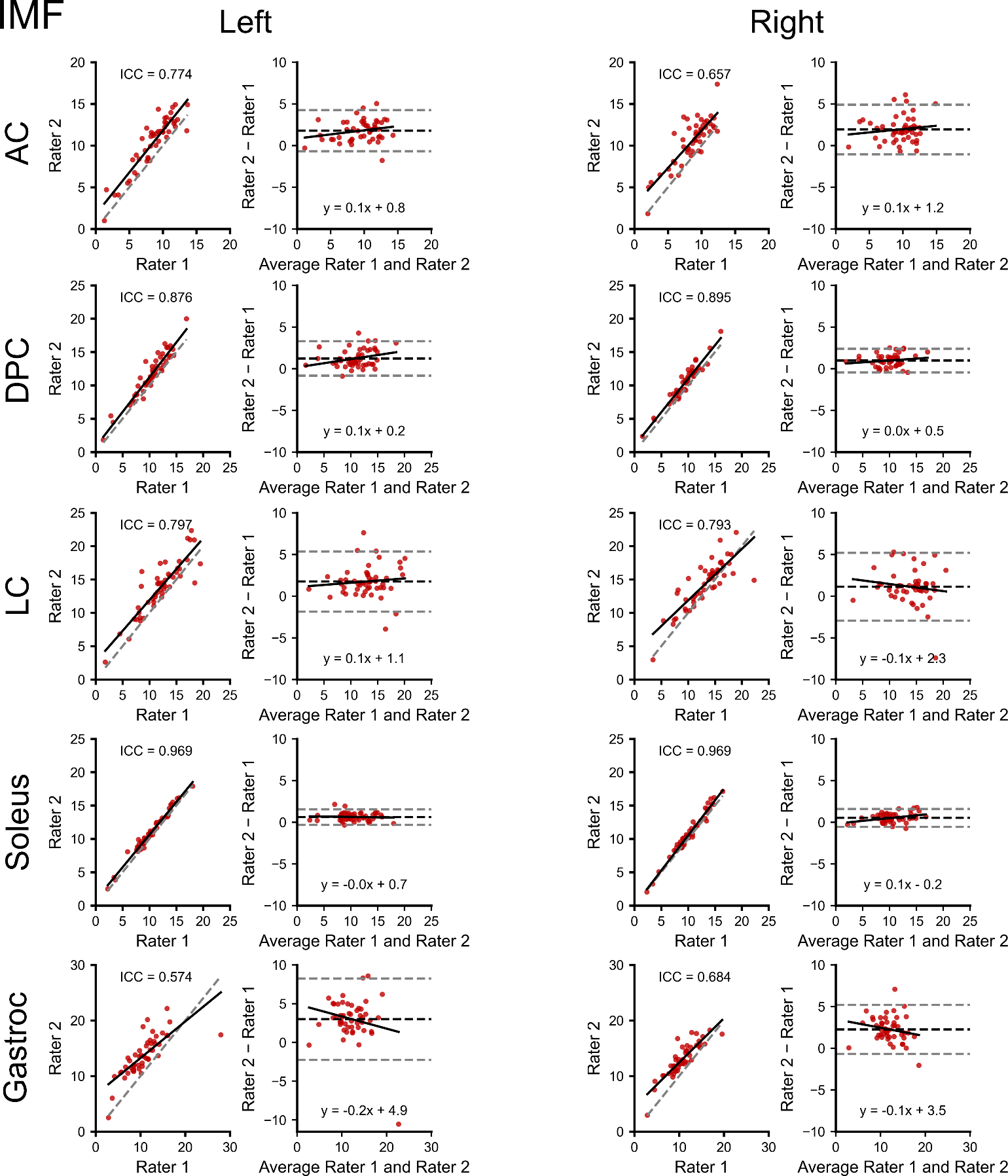


**Supplementary Figure 2.** Interrater accuracy and reliability of intramuscular fat (IMF, %) between the two manual raters on the training and testing datasets (n = 50) without eroding the segmentations. Correlation and Bland-Altman plots are shown for each muscle. In the correlation plot, the solid black line represents the best fit line, and the dashed gray line represents perfect agreement (y = x). In the Bland-Altman plots, the dashed black and gray lines indicate the mean difference (i.e., bias) ± 1.96 × standard deviation (i.e., 95% limits of agreement). The solid black line together with the linear regression equation summarize the direction and magnitude of proportional bias. ICC = intraclass correlation coefficient. AC = anterior compartment, DPC = deep posterior compartment, LC = lateral compartment, Gastroc = gastrocnemius.


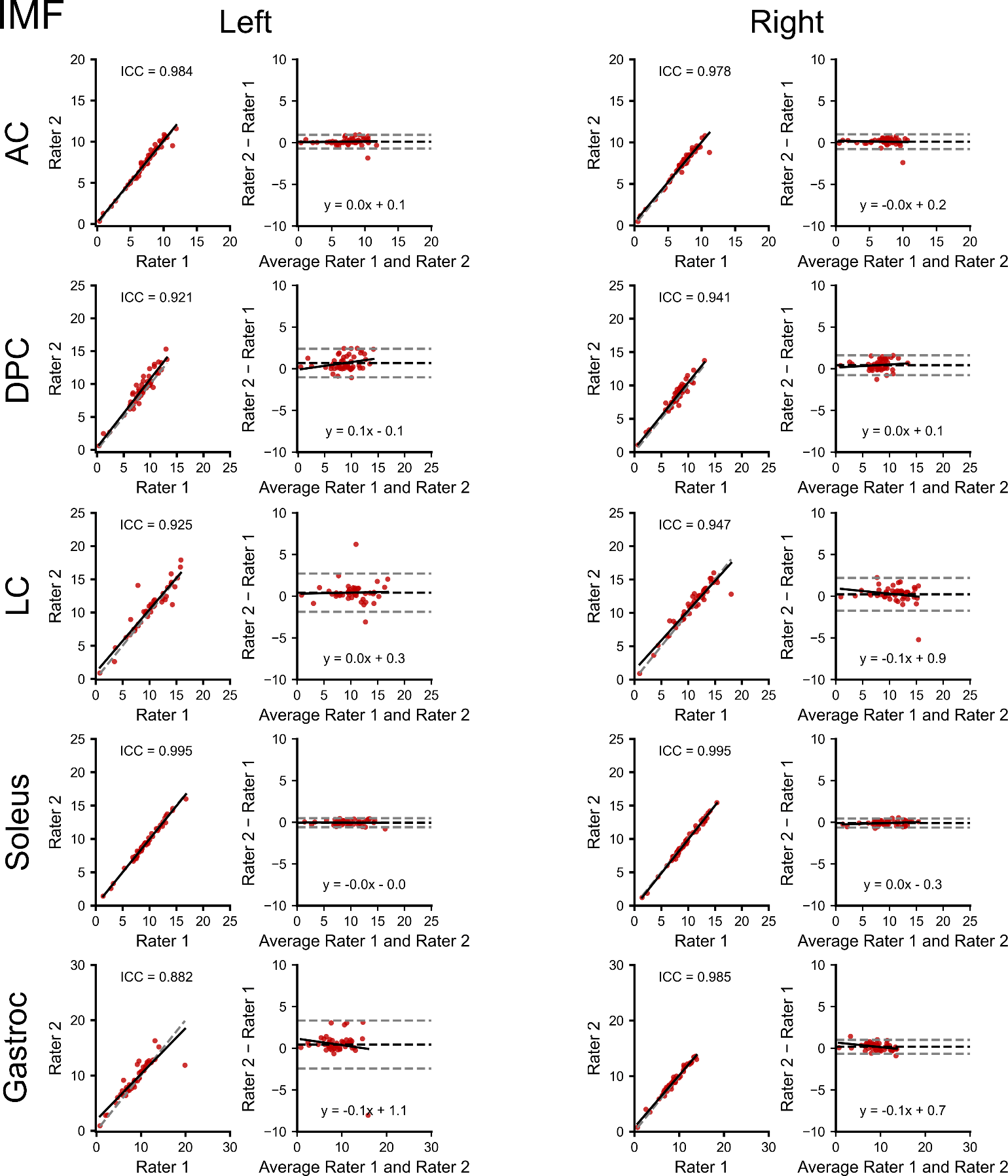


**Supplementary Figure 3.** Interrater accuracy and reliability of intramuscular fat (IMF, %) between the two manual raters on the training and testing datasets (n = 50) after eroding the segmentations. Correlation and Bland-Altman plots are shown for each muscle. In the correlation plot, the solid black line represents the best fit line, and the dashed gray line represents perfect agreement (y = x). In the Bland-Altman plots, the dashed black and gray lines indicate the mean difference (i.e., bias) ± 1.96 × standard deviation (i.e., 95% limits of agreement). The solid black line together with the linear regression equation summarize the direction and magnitude of proportional bias. ICC = intraclass correlation coefficient. AC = anterior compartment, DPC = deep posterior compartment, LC = lateral compartment, Gastroc = gastrocnemius.

**Supplementary Table 4. Testing Segmentation Accuracy (n = 16)**

| **Muscle** | **Side** | **Dice** | **JI** | **CC** | **TPR** | **TNR** | **PPV** | **VR** |
| --- | --- | --- | --- | --- | --- | --- | --- | --- |
| AC | Left | 0.93 (< 0.01) | 0.87 (< 0.01) | 0.85 (0.01) | 0.94 (< 0.01) | 1.00 (< 0.01) | 0.92 (0.01) | 1.03 (0.01) |
|  | Right | 0.93 (< 0.01) | 0.87 (< 0.01) | 0.85 (< 0.01) | 0.93 (< 0.01) | 1.00 (< 0.01) | 0.93 (< 0.01) | 1.01 (0.01) |
| DPC | Left | 0.87 (0.01) | 0.78 (0.01) | 0.71 (0.01) | 0.90 (0.01) | 1.00 (< 0.01) | 0.85 (0.01) | 1.06 (0.02) |
|  | Right | 0.87 (0.01) | 0.78 (0.01) | 0.71 (0.02) | 0.89 (0.01) | 1.00 (< 0.01) | 0.86 (0.01) | 1.04 (0.01) |
| LC | Left | 0.90 (0.01) | 0.82 (0.01) | 0.78 (0.02) | 0.91 (0.01) | 1.00 (< 0.01) | 0.90 (0.01) | 1.01 (0.02) |
|  | Right | 0.90 (< 0.01) | 0.82 (0.01) | 0.77 (0.01) | 0.89 (0.01) | 1.00 (< 0.01) | 0.91 (0.01) | 0.98 (0.02) |
| Soleus | Left | 0.93 (< 0.01) | 0.86 (< 0.01) | 0.84 (0.01) | 0.94 (< 0.01) | 1.00 (< 0.01) | 0.92 (0.01) | 1.02 (0.01) |
|  | Right | 0.92 (< 0.01) | 0.86 (0.01) | 0.84 (0.01) | 0.93 (< 0.01) | 1.00 (< 0.01) | 0.92 (0.01) | 1.02 (0.01) |
| Gastroc | Left | 0.89 (0.01) | 0.80 (0.01) | 0.75 (0.01) | 0.90 (0.01) | 1.00 (< 0.01) | 0.89 (0.01) | 1.02 (0.02) |
|  | Right | 0.89 (0.01) | 0.80 (0.01) | 0.75 (0.01) | 0.91 (0.01) | 1.00 (< 0.01) | 0.87 (0.01) | 1.06 (0.03) |

Performance of the CNN model segmentations were assessed on the testing dataset (n = 16) using the Sørensen-Dice index (Dice), Jaccard index (JI), conformity coefficient (CC), true positive rate (TPR), true negative rate (TNR), positive predictive value (PPV), and volume ratio (VR). Metrics shown = average (standard error). AC = anterior compartment, DPC = deep posterior compartment, LC = lateral compartment, Gastroc = gastrocnemius.

**Supplementary Table 5. Testing Muscle Volume and IMF Accuracy and Reliability (n = 16)**

| **Volume (ml)** | | | | | | | | | |
| --- | --- | --- | --- | --- | --- | --- | --- | --- | --- |
| **Muscle** | **Side** | **Mean** | **Bias** | **95% LA** | **MAE** | **RMSE** | **ICC** | **ICC 95% CI** | **ICC p** |
| AC | Left | 195.6 (13.4) | 3.9 | -11.3 – 19.1 | 7.3 | 8.7 | 0.977 | 0.992 | < 0.001 |
|  | Right | 193.1 (12.9) | 0.4 | -15.1 – 16.0 | 5.3 | 7.9 | 0.981 | 0.994 | < 0.001 |
| DPC | Left | 144.8 (10.0) | 5.9 | -17.1 – 29.0 | 11.9 | 13.2 | 0.912 | 0.967 | < 0.001 |
|  | Right | 142.9 (10.9) | 3.6 | -12.4 – 19.5 | 7.0 | 8.9 | 0.964 | 0.987 | < 0.001 |
| LC | Left | 109.3 (6.2) | 0.3 | -20.0 – 20.5 | 7.0 | 10.3 | 0.846 | 0.920 | < 0.001 |
|  | Right | 106.3 (5.3) | -3.1 | -19.0 – 12.7 | 6.8 | 8.7 | 0.885 | 0.957 | < 0.001 |
| Soleus | Left | 455.2 (22.4) | 7.9 | -25.7 – 41.4 | 15.9 | 18.8 | 0.959 | 0.983 | < 0.001 |
|  | Right | 461.3 (24.0) | 8.7 | -22.4 – 39.8 | 15.7 | 18.1 | 0.963 | 0.986 | < 0.001 |
| Gastroc | Left | 232.2 (18.0) | -1.6 | -43.9 – 40.7 | 17.4 | 21.6 | 0.937 | 0.979 | < 0.001 |
|  | Right | 232.9 (17.8) | 7.5 | -32.9 – 48.0 | 18.5 | 22.0 | 0.925 | 0.970 | < 0.001 |
|  |  |  |  |  |  |  |  |  |  |
| **IMF (%)** | | | | | | | | | |
| **Muscle** | **Side** | **Mean** | **Bias** | **95% LA** | **MAE** | **RMSE** | **ICC** | **ICC 95% CI** | **ICC p** |
| AC | Left | 9.4 (0.8) | -0.5 | -1.7 – 0.8 | 0.6 | 0.8 | 0.927 | 0.977 | < 0.001 |
|  | Right | 9.1 (0.7) | -0.7 | -2.0 – 0.6 | 0.9 | 1.0 | 0.839 | 0.972 | < 0.001 |
| DPC | Left | 10.9 (0.7) | 0.2 | -1.2 – 1.5 | 0.6 | 0.7 | 0.938 | 0.970 | < 0.001 |
|  | Right | 9.5 (0.6) | -0.4 | -1.1 – 0.4 | 0.5 | 0.5 | 0.939 | 0.984 | < 0.001 |
| LC | Left | 12.4 (0.8) | -0.8 | -3.0 – 1.4 | 1.1 | 1.4 | 0.776 | 0.935 | < 0.001 |
|  | Right | 12.4 (0.8) | -1.5 | -3.5 – 0.6 | 1.5 | 1.8 | 0.692 | 0.949 | < 0.001 |
| Soleus | Left | 10.2 (0.8) | -0.4 | -1.1 – 0.2 | 0.5 | 0.6 | 0.970 | 0.995 | < 0.001 |
|  | Right | 10.3 (0.8) | -0.5 | -1.0 – 0.1 | 0.5 | 0.5 | 0.973 | 0.997 | < 0.001 |
| Gastroc | Left | 10.1 (0.8) | -1.9 | -3.9 – 0.1 | 1.9 | 2.2 | 0.549 | 0.951 | < 0.001 |
|  | Right | 10.3 (0.8) | -1.4 | -3.6 – 0.8 | 1.5 | 1.8 | 0.512 | 0.939 | < 0.001 |

Metrics shown = average (standard error)

IMF = intramuscular fat

LA = limits of agreement

MAE = mean absolute error

RMSE = root mean squared error

ICC = intraclass correlation coefficient

CI = Confidence Interval

AC = anterior compartment, DPC = deep posterior compartment, LC = lateral compartment, Gastroc = gastrocnemius.

**Supplementary Table 6. Testing Muscle Volume and IMF Accuracy by Sex**

| **Volume (ml)** | | **Female (n = 8)** | **Male (n = 8)** |  |
| --- | --- | --- | --- | --- |
| **Muscle** | **Side** | **Bias** | **Bias** | **p** |
| AC | Left | 5.6 (2.5) | 2.2 (3.2) | 0.419 |
|  | Right | 4.8 (1.8) | -4 (3.1) | 0.026 |
| DPC | Left | 4 (4.5) | 7.9 (4.2) | 0.540 |
|  | Right | 1.4 (1.7) | 5.7 (3.8) | 0.320 |
| LC | Left | 2.7 (1.7) | -2.2 (5.1) | 0.385 |
|  | Right | 1.4 (2) | -7.7 (2.9) | 0.023 |
| Soleus | Left | 13.9 (5.7) | 1.8 (6.3) | 0.177 |
|  | Right | 12.7 (5.6) | 4.6 (6) | 0.338 |
| Gastroc | Left | 3.1 (6.9) | -6.4 (8.9) | 0.411 |
|  | Right | 9.4 (5.7) | 5.7 (9.4) | 0.746 |
|  |  |  |  |  |
| **IMF (%)** |  | **Female (n = 8)** | **Male (n = 8)** |  |
| **Muscle** | **Side** | **Bias** | **Bias** | **p** |
| AC | Left | < 0.1 (0.1) | < 0.1 (0.1) | 0.519 |
|  | Right | -0.1 (0.1) | -0.3 (0.1) | 0.193 |
| DPC | Left | 0.1 (0.2) | 0.4 (0.2) | 0.331 |
|  | Right | -0.1 (0.1) | < 0.1 (0.1) | 0.605 |
| LC | Left | -0.1 (0.2) | -0.4 (0.4) | 0.525 |
|  | Right | -0.3 (0.1) | -0.4 (0.2) | 0.396 |
| Soleus | Left | < 0.1 (< 0.1) | -0.2 (< 0.1) | 0.005 |
|  | Right | < 0.1 (< 0.1) | -0.1 (< 0.1) | 0.010 |
| Gastroc | Left | -0.6 (0.2) | -0.3 (0.1) | 0.271 |
|  | Right | -0.2 (0.1) | -0.3 (0.1) | 0.463 |

Metrics shown = average (standard error)

IMF = intramuscular fat

AC = anterior compartment, DPC = deep posterior compartment, LC = lateral compartment, Gastroc = gastrocnemius.

**
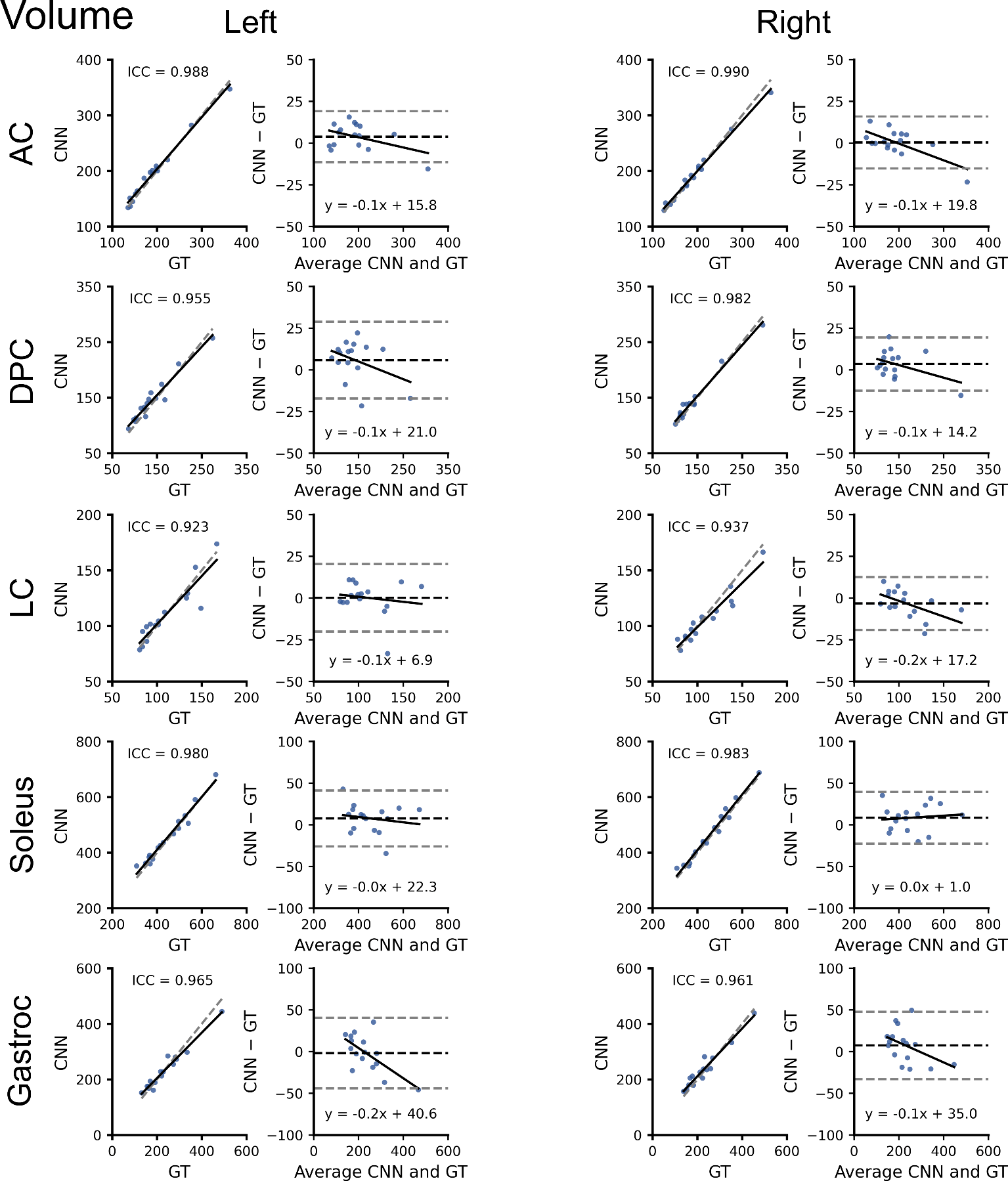
**

**Supplementary Figure 4.** Accuracy and reliability of the automated assessment of muscle volume (ml) with respect to manual segmentation on the testing dataset (n = 16). Correlation and Bland-Altman plots are shown for each muscle. In the correlation plot, the solid black line represents the best fit line, and the dashed gray line represents perfect agreement (y = x). In the Bland-Altman plots, the dashed black and gray lines indicate the mean difference (i.e., bias) ± 1.96 × standard deviation (i.e., 95% limits of agreement). The solid black line together with the linear regression equation summarize the direction and magnitude of proportional bias. ICC = intraclass correlation coefficient, CNN = convolutional neural network, GT = ground truth. AC = anterior compartment, DPC = deep posterior compartment, LC = lateral compartment, Gastroc = gastrocnemius.


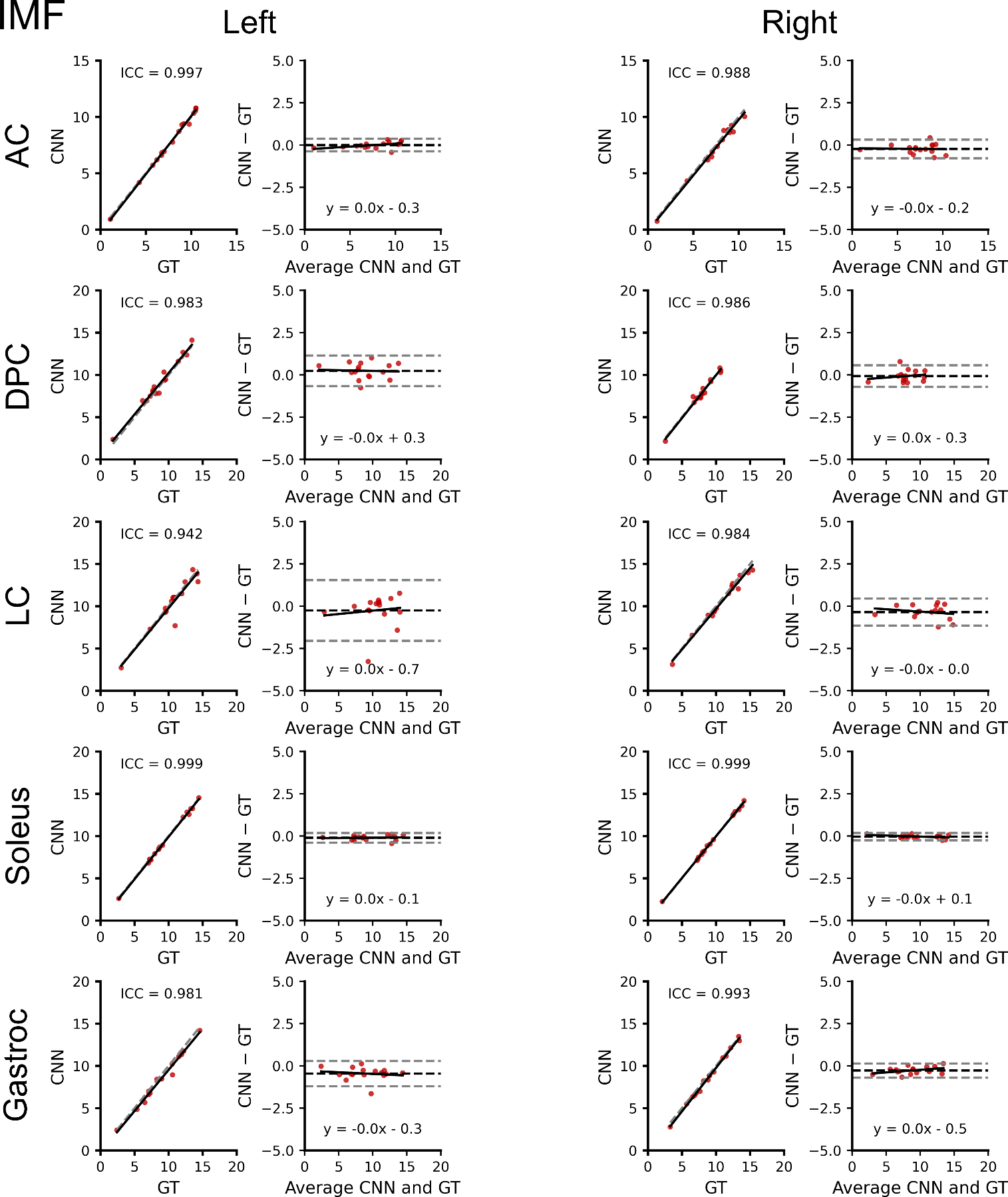


**Supplementary Figure 5.** Accuracy and reliability of the automated assessment of intramuscular fat (IMF, %) with respect to manual segmentation on the testing dataset (n = 16). Correlation and Bland-Altman plots are shown for each muscle. In the correlation plot, the solid black line represents the best fit line, and the dashed gray line represents perfect agreement (y = x). In the Bland-Altman plots, the dashed black and gray lines indicate the mean difference (i.e., bias) ± 1.96 × standard deviation (i.e., 95% limits of agreement). The solid black line together with the linear regression equation summarize the direction and magnitude of proportional bias. ICC = intraclass correlation coefficient, CNN = convolutional neural network, GT = ground truth. AC = anterior compartment, DPC = deep posterior compartment, LC = lateral compartment, Gastroc = gastrocnemius.
